# The application of Foley catheter traction technique in extraperitoneal robot-assisted radical prostatectomy

**DOI:** 10.1186/s12894-023-01377-5

**Published:** 2023-12-05

**Authors:** Xiao-Lu Jiang, Kui OuYang, Rui Yang, Jia-Ning Sun, Feng Zhang, Hong-Wei Zhao

**Affiliations:** 1 Medical College of Qingdao University, Qingdao, Shandong China; 2 Affiliated Hospital of Weifang Medical University, School of Clinical Medicine, Weifang Medical University, Weifang, China; 3 Department of Urology, Affiliated Yantai Yuhuangding Hospital of Qingdao University, NO. 20 East Yuhuangding Road, Yantai, Shandong 264000 China

**Keywords:** Robotic surgery, Prostate cancer, Prostatectomy, Extraperitoneal

## Abstract

**Objectives:**

To describe a technique to improve exposure of prostate during extraperitoneal robot-assisted radical prostatectomy (EP-RARP).

**Material and methods:**

From March 2020 to June 2022, a total of 41 patients with prior intra-abdominal surgery underwent EP-RARP. Twenty-three patients improved exposure by traction of prostate through urinary catheter. The catheter traction prostatectomy (CTP) group was compared with the standard prostatectomy (SP) group using three robotic arms (18 patients) in terms of estimated blood loss (EBL), operative time, positive surgical margin rate, the recovery rate of urinary continence, Gleason score and postoperative hospital stays. Differences were considered significant when *P* < 0.05.

**Results:**

The operative time was lower in the CTP group (109.63 min vs. 143.20 min; *P* < 0.001). EBL in the CTP group was 178.26 ± 30.70 mL, and in the standard prostatectomy group, it was 347.78 ± 53.53 mL (*P* < 0.001). No significant differences with regard to postoperative hospital stay, recovery rate of urinary continence, catheterization time and positive surgical margin were observed between both groups. No intraoperative complications occurred in all the patients. After 6 months of follow-up, the Post-op Detectable prostate specific antigen was similar between the two groups.

**Conclusion:**

CTP is a feasible, safe, and valid procedure in EP-RARP. Application of CTP improved the exposure of prostate, reduced operative time and blood loss in comparison with the conventional procedure.

**Supplementary Information:**

The online version contains supplementary material available at 10.1186/s12894-023-01377-5.

## Introduction

Robot-assisted radical prostatectomy (RARP) has increasingly used worldwide, was the common surgical approach for prostatectomy [1, 2]. The use of minimally invasive techniques reduced the risk of surgical site infection by comparison with open surgery [3]. Prostatectomy has always been led by the transperitoneal approach, due to the larger working space and familiarity with the intra-abdominal landmarks. However, the extraperitoneal approach offers advantages such as: avoiding the peritoneal cavity; less bowel adhesions and ileus; reduced operative time [4, 5]. For patients with prior intra-abdominal surgery, the extraperitoneal approach is a better choice [6]. The extraperitoneal approach has several limitations including a small working space and collision of the robotic arms with one another. The factors resulted in poor prostate exposure in RARP, which ultimately led to increased operative time, intraoperative bleeding and postoperative urinary incontinence rate. We developed a catheter traction technology to improve exposure, which is of great value for prostate resection. In this article, we present this technique and evaluate its feasibility and efficacy in a retrospective case-control comparative study.

## Materials and methods

### Patient selection

From March 2020 to June 2022, 41 patients diagnosed with prostate cancer who had prior abdominal surgery were included in the study. Patients were excluded from this research if they had had any other malignant tumors and serious diseases. All patients were newly diagnosed and had not received other treatments for prostate cancer before, such as brachytherapy, external radiotherapy, chemotherapy, etc. Finally, the catheter traction prostatectomy (CTP) was performed in 23 cases and 18 cases underwent the standard prostatectomy (SP) with three robotic arms. All procedures were performed by the same surgeon. The data of patients’ demographic characteristics, estimated blood loss (EBL), operative time, positive surgical margin rate, the recovery rate of urinary continence, Gleason score and postoperative hospital stays were collected retrospectively. Complications were assessed intraoperatively or postoperatively using the Clavien–Dindo classification system and were classified as major (grade ≥ III) or minor (grade ≤ II ) [7]. All the patients were followed postoperatively. Detailed basic characteristics of patients in each group are summarized in Table 1.

**Table 1:** Preoperative patient characteristics

	CTP group (*n* = 23)	SP group (*n* = 18)	*P* value
Age (years)	66.87 ± 5.98	65.33 ± 8.02	0.486
BMI (Kg/m^2^)	24.83 ± 3.96	24.04 ± 5.03	0.575
PSA (ng/ml)	14.98 ± 8.04	13.11 ± 8.02	0.464
Prostate volume (cc)	50.28 ± 23.90	50.70 ± 26.22	0.958
Biopsy Gleason score (n, %)			0.951
6	10 (43.5)	8 (44.4)	
≥7	13 (56.5)	10 (55.6)	

Data are shown as mean ± SD or n (%)

The study was approved by Affiliated Yantai Yuhuangding Hospital of Qingdao University Ethics Committee. Written informed consent was obtained by the participants. The patients were all informed that their clinical data might be used in future study without invasion of privacy during hospitalization.

### Surgical technique

RARP was performed using the robotic da Vinci Si system. All cases were approached extraperitoneal. A 16-French Foley catheter was placed sterilely after prepping and draping. Patients were placed in a low lithotomy position, and a midline longitudinal skin incision was made 2 cm below the umbilicus. The extraperitoneal space was first established by blunt dissection, with further extension by a handmade balloon. This space was insufflated with CO_2_ gas at a pressure of 12 mmHg. A 12-mm camera was placed through the trocar site. Two 8-mm ports were placed 8 cm away from the umbilicus between the umbilicus and the anterior superior iliac spine. A 12-mm ports were placed above the right anterior superior iliac spines for assistance.

**Fig. 1:**
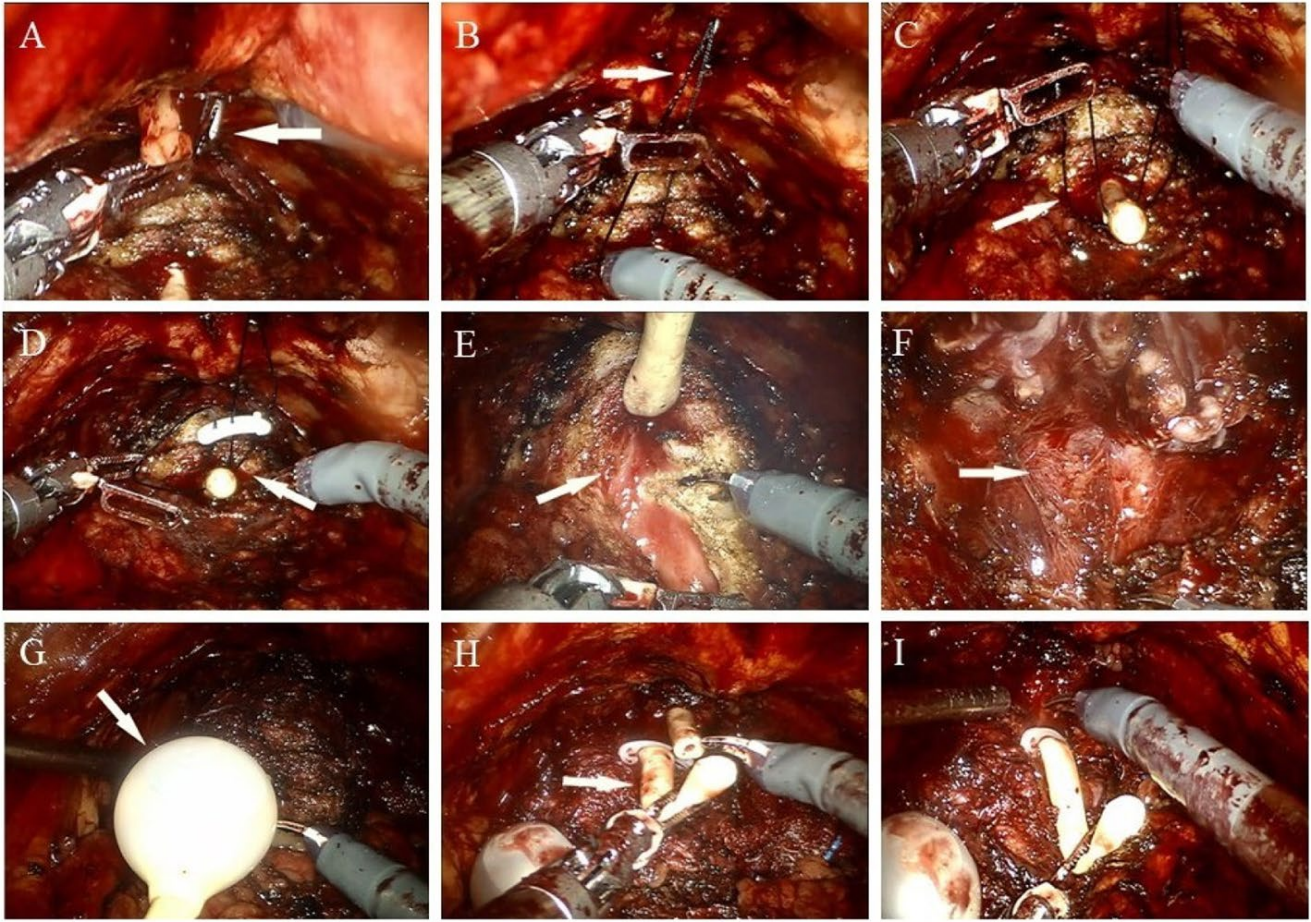
Schematic diagram of the novel technique in EP-RARP. **a**, **b** A syringe needle and suture (white arrow). **c**, **d** The suture was fixed on the catheter (white arrow). **e** The catheter was pulled up (white arrow). **f** Prostate was well exposed (white arrow). **g** The balloon (white arrow) was injected with normal saline. **h**, **i** The catheter (white arrow) was cut off and traction

The prostatic anterior fat pad was removed to define the prostate landmarks. The endopelvic fascia was incised and the deep venous complex was ligated with a 2-0 unidirectional barbed suture. After careful bladder neck dissection, the catheter balloon was emptied. A 0 suture was passed through the abdominal wall with the aid of a 21-gauge syringe needle (Fig. 1a, b). The suture was fixed on the top of the catheter by Hem-o-lok clip (Fig. 1c, d). The tip of the catheter was pulled up through the suture, while the end of the catheter was pulled up to maintain continuous traction on prostate. The exposure of prostate and surrounding structures was significantly improved (Fig. 1e). The dissection of the dorsal prostate and ligaments has become simpler. Carefully dissect the area near the seminal vesicle. The posterior layer of the Denonvillier’s fascia was incised in an antegrade fashion (Fig. 1f). The prostatic pedicles were clipped with Hem-o-lok clip and the neurovascular bundles were dissected off the prostate bluntly if desired. After dissection of the prostatic base, the suture fixed on the top of urinary catheter was cut off.

Then the urethra was also transected at the prostatic apex. After the anterior urethral wall of the apex of the prostate was opened, the catheter balloon was injected with 10 ml of normal saline (Fig. 1g). The catheter was clamped by Hem-o-ok clip to prevent balloon leakage, and then cut off at the distal side of the clip. The catheter was pulled up (Fig. 1h, i). Finally, prostate was pulled upward with residual catheter to improve exposure of the dorsal apex of prostate. Additional movie files show the procedure in more detail (see Additional files 1 and 2).

The specimen was entrapped in an endocatch bag and positioned aside to avoid obstructing the completion of the anastomosis. The vesicourethral anastomosis was performed using 2-0 unidirectional barbed suture in a continuous fashion over a catheter. The specimen was then removed through the extended incision at 12 mm camera port. Extended pelvic lymph node dissection (ePLND) was performed in all patients and the extended template was applied when the risk of lymph node involvement was > 7% according to the Briganti nomogram [8, 9].

### Statistical analysis

The student’s t-test was used to compare continuous variables, and the Chi-square test was used to compare categorical variables. Statistical differences were determined at a *p*-value of < 0.05.

## Results

Patient and tumor characteristics are listed in Table 1. No significant differences were found in patient age, body mass index (BMI), preoperative prostate specific antigen (PSA) level, prostate volume, or Gleason score.

Table 2 shows that catheterization time, positive surgical margin and complications were not significantly different (*P* > 0.05). However, mean operative time was significantly shorter in the CTP group than in the SP group (109.63 vs. 143.20 minutes, *p* < 0.001). And the EBL was lower in the CTP group compared with the SP group (178.26 vs. 347.78, *p* < 0.001). All of the patients underwent a 6-month follow-up, and the 3-month post-op detectable PSA was similar between the two groups. The mean continence rate was slightly higher in the CTP group compared with the SP group, but there was no significant difference. The total traction time was 1.20 ± 0.30 min in the CTP group. No intraoperatively complications occurred in either group. Postoperative pain (Clavien-Dindo classification Gr I) occurred in 1 patient in the CTP group and 2 patients in the SP group, and was relieved after the intervention of flurbiprofen axetil. One patient in the CTP group had postoperative nausea and vomiting (Gr I) and recovered after metoclopramide intervention. One patient in the CTP group underwent urinary tract infection (Gr II) and recovered after 1 week of intervention.

**Table 2:** Intraoperative and postoperative data and complications

	CTP group (*n* = 23)	SP group (*n* = 18)	*P* value
Operative time (min)	109.63 ± 21.05	143.20 ± 29.94	< 0.001
EBL (mL)	178.26 ± 30.70	347.78 ± 53.53	< 0.001
Post-op hospital stays (days)	3.00 ± 1.33	3.89 ± 2.30	0.112
Catheterization time (days)	10.96 ± 2.01	11.89 ± 2.00	0.147
Positive surgical margin (n, %)	8 (34.8)	6 (33.3)	0.923
Pathological T stage (n, %)			0.875
T2a	3 (13.0)	2 (11.1)	
T2b	1 (4.3)	2 (11.1)	
T2c	8 (34.8)	6 (33.3)	
T3a	11 (47.9)	8 (44.5)	
Post-op complications (n, %)			
Clavien I–II	3 (13.0)	2 (11.1)	0.851
Clavien III–V	0 (0)	0 (0)	0 (0)
3 months post-op detectable PSA (n, %)	1 (4.3)	0 (0)	0.370
6 months recovery rate of urinary continence (n, %)	20 (86.9)	14 (77.7)	0.438
Cost of robotic surgery (¥)	40,575.49 ± 1486.83	34,737.88 ± 1762.79	<0.001

Data are shown as mean ± SD or n (%)

## Discussion

RARP has become an important treatment choice for localized prostate cancer and is regarded as the standard surgical approach intreating localized prostate cancer. Previous meta-analyses have shown the advantages of RARP including lower blood transfusion rate, better urinary continence recovery and better potency rate after surgery [10–12]. Although the transperitoneal approach in RARP remains popular at present, the extraperitoneal approach may offer certain advantages in terms of reduced intraperitoneal complications and thus shorten the discharge time [13–17]. Extraperitoneal approach offers faster operative time, decreased length of post-operative stay, and decreased rates of post-operative ileus and inguinal hernia formation [13, 18]. Extraperitoneal approach was a better choice for patients who have previously undergone intra-abdominal surgery [6, 19]. However, the extraperitoneal approach had smaller operative space than transperitoneal approach, which limited the number of mechanical arms in the extraperitoneal approach.

Esposito et al. used an external mechanical device to replace the fourth arm, which reduced medical costs eliminated the need for a dedicated bedside second assistant [20]. However, it was still a challenge to use the device to maintain the robot’s vision and avoid robot arm collision, especially for patients with small body. We developed a simpler method to replace the fourth arm, which reduces medical costs and avoids narrowing of the extraperitoneal space. In this study, the Foley catheter exerted traction on the prostate during prostate resection. It was important to dissect the seminal vesicles completely before opening the posterior layer of the Denonvillier’s fascia. In RARP, dealing with well exposed seminal vesicles could reduce the operative difficulty. And excellent exposure of Denonvillier’s fascia and lateral ligaments could also be attained after the prostate was elevated in the direction of the symphysis. Improved prostate exposure contributed to finer anatomical and intraoperative hemostasis, resulting in lower EBL in the CTP group. When dissecting the apical prostate, the prostate was pulled up by the catheter, which was particularly helpful in reducing bleeding. The apical dissection was one of most crucial and difficult parts of the procedure [21]. Moreover, CTP avoided a limited space caused by increasing the mechanical arm, which was conducive to maintain the robot’s vision. The study indicates that operative time and EBL were significantly reduced using CTP. The operative time and amount of bleeding are very important when considering the feasibility and safety of an operative. A recent high-volume surgical center experience showed that the average operative time and average EBL of conventional extraperitoneal RARP were 146 min and 100 ml, respectively [22]. Ploussard et al. reported that the median operative time and median EBL of RARP performed using an extraperitoneal approach were 128.9 min and 515.4 ml, respectively [23]. The study showed that the operative time and EBL in the CTP group were 109.63 min and 178.26 ml, respectively. The fixed traction delivered by the device served the same function as the fourth robot arm, but it’s not as convenient and flexible as a robotic arm, which extends the time spent on ePLND. Although it took a little time for the prostate to be pulled up, the improvement of exposure saved more time. With the approach, the prostate was fully exposed without adding an additional mechanical arm or external mechanical device. Compared to the expensive cost of robotic arms, catheter traction technology reduces medical costs by nearly a thousand dollars. Improvement of intraoperative prostate exposure was beneficial to reconstruction. Urethral anatomical reconstruction technology played an important role in the early recovery of urinary continence [24–26]. However, according to the functional follow-up results obtained 6 months postoperatively, the recovery rate of urinary continence was similar between the two groups. The result may be caused by insufficient sample size.

Our study has several limitations. First, this was not a prospective analysis. Second, this was a single-center retrospective study. The sample size was small, and subsequent studies are needed to confirm long-term follow-up data.

## Conclusion

Catheter traction technique offers a simple, inexpensive tool to reproduce the traction provided by the fourth arm. Use of a catheter requires no additional equipment and has aided better exposure of prostate.

### Supplementary Information

**Additional file 1.** The exposure of the dorsal prostate

**Additional file 2.** The exposure of the dorsal apex of prostate

## Data Availability

The data that support the findings of this study are available from the corresponding author upon reasonable request.

## Abbreviations

EP-RARP: extraperitoneal robot-assisted radical prostatectomy
CTP: catheter traction prostatectomy
BMI: body mass index
SP: standard prostatectomy
EBL: estimated blood loss
BMI: body mass index
PSA: prostate specific antigen
RARP: robot-assisted radical prostatectomy
